# Bleeding Risk Prediction in Patients Treated with Antithrombotic Drugs According to the Anatomic Site of Bleeding, Indication for Treatment, and Time Since Treatment Initiation

**DOI:** 10.1055/a-2259-1134

**Published:** 2024-03-18

**Authors:** Vinai Bhagirath, Tanya Kovalova, Jia Wang, Lizhen Xu, Shrikant I. Bangdiwala, Martin O'Donnell, Ashkan Shoamanesh, Jackie Bosch, Rosa Coppolecchia, Tatsiana Vaitsiakhovich, Frank Kleinjung, Hardi Mundl, John Eikelboom

**Affiliations:** 1 Population Health Research Institute, Hamilton, Ontario, Canada; 2 McMaster University, Hamilton, Ontario, Canada; 3 University of Galway, Galway, Galway, Ireland; 4 Bayer US LLC, Whippany, New Jersey, United States; 5 Bayer AG, Berlin, Germany; 6 Bayer AG, Wuppertal, Germany

**Keywords:** bleeding, risk prediction, antithrombotic, atrial fibrillation, ESUS, CAD, PAD

## Abstract

**Background** Reasons for the relatively poor performance of bleeding prediction models are not well understood but may relate to differences in predictors for various anatomical sites of bleeding.

**Methods** We pooled individual participant data from four randomized controlled trials of antithrombotic therapy in patients with coronary and peripheral artery diseases, embolic stroke of undetermined source (ESUS), or atrial fibrillation. We examined discrimination and calibration of models for any major bleeding, major gastrointestinal (GI) bleeding, and intracranial hemorrhage (ICH), according to the time since initiation of antithrombotic therapy, and indication for antithrombotic therapy.

**Results** Of 57,813 patients included, 1,948 (3.37%) experienced major bleeding, including 717 (1.24%) major GI bleeding and 274 (0.47%) ICH. The model derived to predict major bleeding at 1 year from any site (c-index, 0.69, 95% confidence interval [CI], 0.68–0.71) performed similarly when applied to predict major GI bleeding (0.71, 0.69–0.74), but less well to predict ICH (0.64, 0.61–0.69). Models derived to predict GI bleeding (0.75, 0.74–0.78) and ICH (0.72, 0.70–0.79) performed better than the general major bleeding model. Discrimination declined over time since the initiation of antithrombotic treatment, stabilizing at approximately 2 years for any major bleeding and major GI bleeding and 1 year for ICH. Discrimination was best for the model predicting ICH in the ESUS population (0.82, 0.78–0.92) and worst for the model predicting any major bleeding in the coronary and peripheral artery disease population (0.66, 0.65–0.69).

**Conclusion** Performance of risk prediction models for major bleeding is affected by site of bleeding, time since initiation of antithrombotic therapy, and indication for antithrombotic therapy.

## antithrombotic therapy Introduction

Bleeding is the most common adverse event in patients treated with antithrombotic therapy and is independently associated with the risk of subsequent morbidity and mortality. 1 Numerous bleeding risk prediction models have been proposed for clinical use, but most poorly discriminate risk and do not predict the risk for individual patients accurately enough to guide management decisions. 2 Reasons for their poor performance are not well understood, but there are several possible explanations. Risk factors for bleeding differ by anatomical sites related to vascular bed-specific hemostasis, 3 but most models do not distinguish between bleeding at different sites. The risk of bleeding changes over time but most models do not consider risk in relation to the timing of commencement of antithrombotic therapy. Most bleeding models have been developed in selected patient populations and antithrombotic regimens and may not perform as well when applied to other populations.

To further explore the determinants of bleeding risk prediction, we analyzed pooled individual patient data from four large randomized controlled trials of antithrombotic therapy in patients with coronary and peripheral artery diseases, embolic stroke of undetermined source (ESUS), or atrial fibrillation, in which bleeding events were systematically collected. Our objectives were to investigate the impacts of the site of bleeding, timing of bleeding in relation to the commencement of antithrombotic therapy, and indication for antithrombotic therapy on bleeding risk prediction.

## Methods

### Dataset

We combined data from the Cardiovascular Outcomes for People Using Anticoagulation Strategies (COMPASS), New Approach Rivaroxaban Inhibition of Factor Xa in a Global Trial versus acetyl salicylic acid (ASA) to Prevent Embolism in Embolic Stroke of Undetermined Source (NAVIGATE ESUS), Randomized Evaluation of Long-Term Anticoagulation Therapy (RE-LY), and Apixaban Versus Acetylsalicylic Acid to Prevent Stroke in Atrial Fibrillation Patients Who Have Failed or Are Unsuitable for Vitamin K Antagonist Treatment (AVERROES) randomized trials that compared various antithrombotic strategies in patients with coronary and peripheral artery diseases, ESUS, or atrial fibrillation. 4 5 6 7 We included trials of antithrombotic agents, for which we had access to individual participant-level data and which collected bleeding outcomes. A summary of the trial designs is provided in Supplementary Table S1 (available in the online version).

### Baseline Predictors of Bleeding

A list of baseline variables reported in the trials is provided in Supplementary Table S2 (available in the online version). Variables that were included in one or more previously published risk scores or felt to be relevant, and which were collected in all four studies, were considered as candidate variables when building the model.

### Outcome Definitions

Outcome events reported in the RE-LY and AVERROES trials were adjudicated by an independent panel who were blinded to the treatment assignment. Outcome events reported in the COMPASS and NAVIGATE ESUS trials were first screened by a computer algorithm and only those that did not clearly meet protocol definitions were adjudicated by a blinded panel. 8 We included bleeding as defined in each trial and did not attempt to reclassify events.

#### Major Bleeding

The definition of major bleeding in all four trials was closely aligned with the International Society on Thrombosis and Haemostasis definition and included fatal bleeding; symptomatic bleeding in a critical area or organ, such as intracranial, intraspinal, intraocular, retroperitoneal, intra-articular or pericardial, or intramuscular with compartment syndrome; and/or bleeding causing a fall in the hemoglobin level of 20 g/L or more, or leading to the transfusion of two or more units of whole blood or red cells. The only exception was the COMPASS trial in which bleeding resulting in a visit to an acute care medical facility replaced a 20 g/L hemoglobin drop in the definition for major bleeding.

#### Major Gastrointestinal Bleeding

Each trial defined major gastrointestinal (GI) bleeding as major bleeding from any GI source.

#### Intracranial Hemorrhage

ICH was defined as bleeding that was intracranial and included subdural, epidural, subarachnoid, and intraparenchymal bleeding. Hemorrhagic transformation of ischemic stroke was not included in the definition of ICH.

#### Major Adverse Cardiovascular Events

Major adverse cardiac events (MACEs) included myocardial infarction, ischemic stroke, and cardiovascular death. The definition of myocardial infarction in all studies adhered to the Third Universal Definition of Myocardial Infarction. 9 Ischemic stroke was defined as the presence of acute focal neurological deficit thought to be of vascular origin with signs and symptoms lasting ≥ 24 hours or to the time of death, with computed tomography or magnetic resonance imaging or autopsy that does not show primary hemorrhage (although hemorrhagic transformation is consistent with ischemic stroke). In the NAVIGATE ESUS trial, a deficit lasting less than 24 hours was considered ischemic stroke if evidence of acute brain infarct was present on neuroimaging. Cardiovascular death was any death for which no definite noncardiovascular cause could be identified.

### Statistical Analyses

We developed separate Cox proportional hazards models, stratified by trial, for the prediction of major bleeding from any anatomical site, major GI bleeding, and ICH. We compared the discriminative performance of these models in predicting bleeding from different anatomical sites, according to durations of follow-up after the initiation of antithrombotic treatment, and in patients with different indications for antithrombotic therapy.

#### Model Building

Study-specific Cox proportional hazard models were applied to the time-to-event analysis, and the multivariable fractional polynomial algorithm 10 was employed to both select covariates for the model and choose the functional form of the continuous covariates if their effects on the dependent variable were nonlinear. This method does not require the categorization of continuous covariates, even when they do not have a linear relationship with the outcome of interest. Competing risk analysis was performed using the Fine and Gray method 11 ; MACE during follow-up was considered as a competing risk for major bleeding, and MACE and major bleeding at another site were considered as competing risks for major GI bleeding and ICH, respectively. Patients with missing values were excluded. For variables deemed multicollinear due to correlation matrix value ≥0.4, or a variance inflation factor ≥10, the variable identified by the model-building algorithm as having greater predictive utility was selected for the covariate candidacy set. The proportional hazards assumption was checked by Schoenfeld residuals, Cox regression to assess for interaction with time, and visual assessment of log–log survival plots.

#### Assessment of Confounders

Each excluded variable was added one-by-one back to the model, and the change in coefficients for all selected variables was assessed. Confounders were considered to exist if, by including the confounding variable in the regression model, there was a change of 10% or greater in the coefficient of any other variable in the model. 12

#### Assessment of Model Performance

Model discrimination was assessed using Harrell's c-index weighted over four strata defined by the individual study populations. 13 Continuous calibration curves were produced using restricted cubic splines, and the integrated calibration index (the mean absolute difference between observed and predicted probabilities, weighted by the distribution of predicted probabilities) was calculated. 14 To assess how the discriminative performance changed depending on the length of follow-up for the bleeding outcomes, we truncated follow-up at varying times and calculated the c-index for each time point. 13 For internal validation, 200 bootstrap samples were created using the random selection of participants, with replacement. An optimism-corrected c-index was calculated using these samples. 15

#### Comparison of Predictors Between Site-Specific Models

A list was composed of all variables that were selected in any of the three models (major bleeding, major GI bleeding, and ICH). These variables were then forced into two new multivariable fractional polynomial models to predict major GI bleeding and ICH. Variables whose hazard ratios had nonoverlapping 95% confidence intervals (CIs) were deemed of interest as potentially having different predictive abilities for the two sites of bleeding.

## Results

### Characteristics of Included Patients

Of 57,813 patients enrolled in the four trials, 57,383 had complete data for the set of considered covariates and were included in the model-building analyses. Median age was 70 years (interquartile range [IQR]: 65–75), and 30.3% were female. Study antithrombotic treatment was full-intensity direct oral anticoagulant (DOAC)—dabigatran 150 or 110 mg bid, apixaban 5 mg bid or 2.5 mg in patients meeting at least two of three criteria for dose reduction [age ≥ 80 years, weight ≤ 60 kg, creatinine ≥ 133 µmol/L], or rivaroxaban 15 mg od, in 18,216 (31.5%); warfarin in 5,879 (10.2%); aspirin (81 to 324 mg od) alone in 15,475 (26.8%); rivaroxaban 2.5 bid plus aspirin in 9,135 (15.8%), and rivaroxaban 5 mg bid in 9,108 (15.8%). Table 1 describes the baseline characteristics in the overall patient population and separately in those with no major bleeding, major bleeding at any site, major GI bleeding, and ICH.

**Table 1:** Baseline characteristics of included patients

Variable	Overall ( *N* = 57,813)	Major bleeding ( *N* = 1,948)	No major bleeding ( *N* = 55,865)	Major GI bleeding ( *N* = 717)	Intracranial bleeding ( *N* = 274)
Randomized treatment arm					
Aspirin	15,475 (26.8)	222 (11.4)	15,248 (27.3)	77 (10.7)	46 (16.8)
Warfarin	5,879 (10.2)	404 (20.7)	5,472 (9.8)	108 (15.1)	78 (28.5)
DOAC, therapeutic dose	18,216 (31.5)	793 (40.7)	17,409 (31.2)	313 (43.7)	82 (29.9)
Rivaroxaban alone	9,108 (15.8)	246 (12.6)	8,862 (15.9)	88 (12.3)	39 (14.2)
Rivaroxaban + Aspirin	9,135 (15.8)	283 (14.5)	8,851 (15.9)	131 (18.3)	29 (10.6)
Age (years)	70.0 (65.0, 75.0)	74.0 (69.0, 79.0)	69.0 (65.0, 75.0)	75.0 (69.0, 79.0)	74.0 (68.0, 79.0)
Sex: Female	17,518 (30.3)	582 (29.9)	16,936 (30.3)	209 (29.1)	79 (28.8)
Race					
Asian	9,550 (16.5)	300 (15.4)	9,250 (16.6)	116 (16.2)	77 (28.1)
Black/African American	579 (1.0)	26 (1.3)	553 (1.0)	11 (1.5)	4 (1.5)
White/Caucasian	37,796 (65.4)	1,361 (69.9)	36,435 (65.2)	477 (66.5)	161 (58.8)
Other	9,886 (17.1)	261 (13.4)	9,625 (17.2)	113 (15.8)	32 (11.7)
Weight (kg)	79.3 (69.0, 90.7)	80.0 (68.2, 91.6)	79.2 (69.0, 90.7)	79.2 (69.0, 90.7)	75.0 (66.0, 85.0)
Height (cm)	169.0 (162.0, 175.3)	170.0 (162.0, 176.0)	169.0 (162.0, 175.3)	169.0 (162.0, 175.3)	169.4 (162.0, 176.0)
BMI	27.7 (24.8, 31.1)	27.8 (24.6, 31.1)	27.7 (24.8, 31.1)	27.7 (24.8, 31.1)	26.1 (23.6, 29.7)
Hip circumference a (cm)	104.0 (97.0, 111.0)	104.1 (98.0, 112.0)	104.0 (97.0, 111.0)	104.0 (98.0, 111.8)	102.0 (96.0, 109.0)
Waist circumference a (cm)	100.0 (91.4, 109.0)	101.0 (91.4, 110.0)	100.0 (91.4, 109.0)	100.0 (91.0, 110.0)	98.5 (90.0, 106.7)
Waist-to-hip ratio a	1.0 (0.9, 1.0)	1.0 (0.9, 1.0)	1.0 (0.9, 1.0)	1.0 (0.9, 1.0)	1.0 (0.9, 1.0)
Baseline diastolic blood pressure	79.0 (70.0, 84.0)	75.0 (68.5, 82.0)	79.0 (70.0, 84.5)	72.5 (67.3, 80.0)	78.0 (70.0, 84.0)
Baseline systolic blood pressure	132.0 (121.0, 144.0)	131.0 (120.0, 144.0)	132.0 (121.0, 144.0)	130.8 (120.0, 144.0)	134.0 (120.0, 145.5)
Pulse pressure	55.0 (46.0, 64.5)	56.0 (47.0, 67.0)	55.0 (46.0, 64.0)	57.0 (48.0, 69.0)	55.0 (46.0, 64.0)
Heart rate	70.0 (62.0, 78.0)	70.0 (62.0, 80.0)	70.0 (61.0, 78.0)	70.0 (61.5, 78.0)	70.0 (63.0, 78.0)
Creatinine (µmol/L)	88.0 (74.0, 104.0)	96.0 (80.0, 115.0)	88.0 (73.0, 103.0)	97.0 (80.0, 115.0)	88.4 (79.0, 106.1)
GFR (mL/min per 1.73m ^2^ )	73.1 (58.4, 88.6)	64.0 (50.0, 81.3)	73.5 (59.0, 88.7)	62.9 (48.2, 78.5)	65.0 (51.6, 79.0)
PAD	8,387 (14.5)	272 (14.0)	8,115 (14.5)	105 (14.6)	24 (8.8)
Stroke	4,708 (8.1)	202 (10.4)	4,506 (8.1)	62 (8.6)	52 (19.0)
TIA	2,949 (5.1)	145 (7.4)	2,804 (5.0)	53 (7.4)	18 (6.6)
Hypertension	44,950 (77.8)	1,576 (80.9)	43,374 (77.6)	582 (81.2)	221 (80.7)
MI a	20,242 (35.0)	673 (34.5)	19,569 (35.0)	272 (37.9)	84 (30.7)
Heart failure	13,937 (24.1)	522 (26.8)	13,415 (24.0)	213 (29.7)	61 (22.3)
Atrial fibrillation b	23,253 (40.2)	1,176 (60.4)	22,077 (39.5)	414 (57.7)	150 (54.7)
Coronary artery disease a	30,194 (52.2)	1,054 (54.1)	29,140 (52.2)	413 (57.6)	127 (46.4)
Cancer	4,568 (7.9)	249 (12.8)	4,319 (7.7)	82 (11.4)	31 (11.3)
Diabetes	17,315 (30.0)	610 (31.3)	16,705 (29.9)	226 (31.5)	74 (27.0)
Prerandomization medication use					
ACE or ARB	37,945 (65.6)	1,327 (68.1)	36,618 (65.5)	499 (69.6)	171 (62.4)
Calcium channel blocker	16,113 (27.9)	624 (32.0)	15,489 (27.7)	225 (31.4)	82 (29.9)
Beta blocker	34,505 (59.7)	1,206 (61.9)	33,299 (59.6)	442 (61.6)	154 (56.2)
Diuretic	21,017 (36.4)	927 (47.6)	20,090 (36.0)	362 (50.5)	92 (33.6)
Statin	38,739 (67.0)	1,220 (62.6)	37,519 (67.2)	466 (65.0)	155 (56.6)
Antiplatelet b	44,077 (76.2)	1,350 (69.3)	42,727 (76.5)	503 (70.2)	197 (71.9)
Anticoagulant b	8,488 (14.7)	460 (23.6)	8,028 (14.4)	162 (22.6)	62 (22.6)
PPI b	14,083 (24.4)	507 (26.0)	13,576 (24.3)	194 (27.1)	70 (25.5)
NSAID	3,060 (5.3)	146 (7.5)	2,914 (5.2)	60 (8.4)	20 (7.3)
>12 years education a	17,759 (30.7)	369 (18.9)	17,390 (31.1)	132 (18.4)	66 (24.1)
Smoking					
Never	24,307 (42.0)	719 (36.9)	23,588 (42.2)	251 (35.0)	111 (40.5)
Former	24,459 (42.3)	1,003 (51.5)	23,456 (42.0)	381 (53.1)	129 (47.1)
Current	9,041 (15.6)	226 (11.6)	8,815 (15.8)	85 (11.9)	34 (12.4)
> 5 drinks of alcohol/wk a	16,975 (29.4)	635 (32.6)	16,340 (29.2)	221 (30.8)	83 (30.3)

Abreviations: ACE, angiotensin-converting enzyme; ARB, angiotensin II receptor blockers; BMI, body mass index; DOAC, direct oral anticoagulant; GFR, glomerular filtration rate; MI, myocardial infarction;NSAID, nonsteroidal anti-inflammatory drugs; PAD, peripheral artery disease; PPI, proton-pump inhibitors; TIA, transient ischemic attack;

Note: Patients categorized according to first major bleeding event. Continuous variables are summarized as median (quartile 1, quartile 3). Categorical variables are summarized as frequency (percent).

a Excluded from model building as variable not collected in all trials.

b Excluded from model building because variable represents prerandomization use of a medication which was randomized in a trial, or an inclusion/exclusion criterion of a trial.

### Events Included

In the competing risk analysis, there were 1,948 (3.37%) patients whose first event was a major bleed, 717 (1.24%) whose first event was a major GI bleed, and 274 (0.47%) whose first event was an intracranial bleed. The number of patients with a bleeding event in each of these categories is summarized by trial in Supplementary Table S3 (available in the online version).

### Variables Selected in the Models

Thirty-eight variables were identified as potentially being of interest. Of these, four were excluded from the models since they represented prerandomization use of medications whose subsequent use was randomized (prerandomization use of anticoagulant, antiplatelet agent, or proton pump inhibitor), or they represented inclusion or exclusion criteria for a trial (history of atrial fibrillation), thereby limiting the interpretability of their relationship to outcomes. An additional six variables were excluded because they were not collected in all four studies (see Table 1 ). The remaining 28 were used for developing the models ( Table 1 ). Supplementary Table S4 (available in the online version) displays hazard ratios associated with each variable in univariate analysis, for the outcomes of major bleeding, major GI bleeding, and ICH respectively.

In total, 17 variables were selected for the major bleeding model, 12 for the major GI bleeding model, and 8 for the ICH model. Randomized treatment arm, age, Asian race, history of hypertension, smoking history, and baseline use of diuretics were selected in all three models. Sex was deemed of interest a priori and was forced into all models. The only confounder according to our criteria was heart rate, which resulted in an 18% decrease in the coefficient for female sex, only in the major GI bleeding model. Table 2 displays these models, with adjusted hazard ratios for each variable selected in the respective models.

**Table 2:** Fractional polynomial models and adjusted hazard ratios (95% confidence intervals) of included variables for major bleeding, major GI bleeding, and intracranial hemorrhage

Variable name	Major bleeding	Major GI bleeding	Intracranial hemorrhage
Randomized treatment arm			
Warfarin vs. aspirin	1.88 (1.33–2.65)	0.93 (0.51–1.68)	5.18 (2.67–10.03)
DOAC, therapeutic dose vs. aspirin	1.58 (1.15–2.18)	1.19 (0.68–2.08)	1.60 (0.92–2.79)
Rivaroxaban alone vs. aspirin	1.57 (1.29–1.92)	1.70 (1.21–2.39)	1.49 (0.91–2.45)
Rivaroxaban plus aspirin vs. aspirin	1.77 (1.46–2.15)	2.45 (1.78–3.37)	1.10 (0.65–1.87)
Baseline biometrics			
Age (per 10 units increase)	1.68 (1.57–1.80)	1.94 (1.74–2.17)	1.78 (1.52–2.09)
Female	1.01 (0.90–1.14)	1.02 (0.84–1.24)	0.86 (0.64–1.15)
Asian race (vs. non–Asian)	1.14 (0.99–1.31)	1.24 (0.99–1.56)	2.03 (1.55–2.66)
Diastolic blood pressure (per five units increase)	0.94 (0.92–0.96)	0.90 (0.87–0.93)	N/A
Pulse pressure (per five units increase)	1.02 (1.01–1.04)	N/A	N/A
Weight	Selected in transformation a	Selected in transformation a	N/A
Creatinine	Selected in transformation a	Selected in transformation a	N/A
Medical history			
Diabetes	1.13 (1.02–1.25)	N/A	N/A
Cancer	1.19 (1.04–1.37)	N/A	N/A
Heart failure	1.07 (0.96–1.19)	1.22 (1.03–1.45)	N/A
Hypertension	1.16 (1.03–1.30)	1.22 (1.01–1.48)	1.26 (0.93–1.71)
Peripheral artery disease	1.11 (0.96–1.28)	N/A	N/A
Smoking: former vs. never	1.37 (1.23–1.52)	1.50 (1.26–1.78)	1.25 (0.96–1.64)
Smoking: current vs. never	1.52 (1.30–1.79)	1.77 (1.36–2.31)	1.38 (0.92–2.06)
Stroke	1.14 (0.98–1.32)	N/A	2.21 (1.62–3.02)
Prerandomization medication use			
NSAID	1.21 (1.02–1.43)	1.32 (1.01–1.73)	N/A
Diuretic	1.12 (1.02–1.24)	1.22 (1.04–1.43)	0.79 (0.60–1.02)

Abbreviations: DOAC, direct oral anticoagulant; GI, gastrointestinal; NSAID, nonsteroidal anti-inflammatory drug.

Note: Variables/transformations not selected for a specific model are denoted by “N/A”.

a Weight (kg) transformed to (weight) ^−2^ *10 ^4^ and log(weight) in the major bleeding model, and log(weight)*10 and log(weight) ^2^ in the major GI bleeding model. Creatinine (µmol/L) transformed to creatinine ^3^ *(1/10) ^5^ and creatinine ^3^ *log(creatinine)*(1/10) ^5^ in the major bleeding model, and creatinine ^3^ *(1/10 ^5^ ) and creatinine ^3^ *log(creatinine)*(1/10 ^5^ ) in the major GI bleeding model. Adjusted hazard ratios for transformed variables not shown due to difficulty in interpreting their values.

### Model Discrimination

Discrimination was better for site-specific bleeding models than for the model that predicted major bleeding at any site. Harrell's c-index (95% CI) for the first year of follow-up was 0.69 (0.68–0.71) for the model derived to predict major bleeding at any anatomical site, 0.75 (0.74–0.78) for the model derived to specifically predict major GI bleeding, and 0.72 (0.70–0.79) for the model derived to predict ICH. Optimism-corrected c-indices (95% CI) for 1 year of follow-up were 0.68 (0.67–0.70) for major bleeding, 0.73 (0.72–0.76) for major GI bleeding, and 0.69 (0.67–0.76) for ICH. When the model derived to predict major bleeding from any anatomical site was applied to predict the more specific outcomes of major GI bleeding and ICH respectively, it performed more poorly than the respective site-specific models; c-indices (95% CI) for the major bleeding model at 1 year of follow-up for predicting major GI bleeding and ICH were 0.71 (0.69–0.74) and 0.64 (0.61–0.69; Table 3 )

**Table 3:** Harrell's c-indices and 95% confidence intervals of models derived for prediction of major bleeding, major gastrointestinal bleeding, and intracranial hemorrhage, as applied, unmodified, for prediction of various outcomes at 1 year

Model	Outcome		
	Major bleed	Major GI bleed	ICH
Major bleed	**0.69 (0.68, 0.71)**	0.71 (0.69, 0.74)	0.64 (0.61, 0.69)
	0.68 (0.67, 0.70) a		
Major GI bleed	0.66 (0.64, 0.67)	**0.75 (0.74, 0.78)**	0.60 (0.55, 0.65)
		0.73 (0.72, 0.76) a	
ICH	0.61 (0.57, 0.62)	0.61 (0.56, 0.64)	**0.72 (0.70, 0.79)**
			0.69 (0.67, 0.76) a

Abbreviations: GI, gastrointestinal; ICH, intracranial hemorrhage.

a Optimism-corrected c-index and 95% confidence interval.

**Fig. 1:**
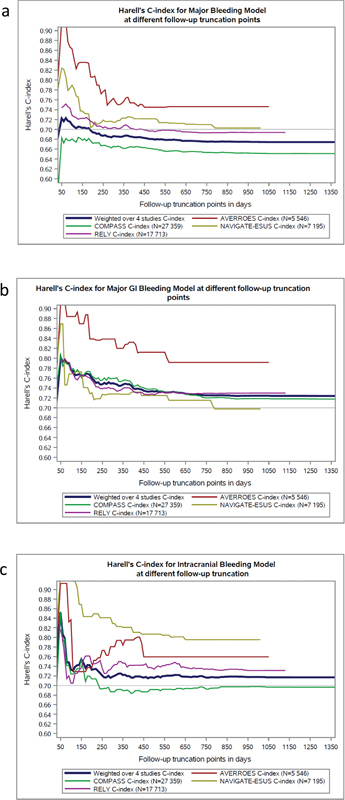
Harrell's c-indices for various follow-up times. ( **a** ) Major bleeding model. ( **b** ) Major GI bleeding model. ( **c** ) Intracranial hemorrhage model.

C-indices were highest within the first 3 months of follow-up, and stabilized after 1 to 2 years of follow-up ( Fig. 1 ). Harrell's c-indices for predicting events that occurred within the first 6 months of follow-up and those that occurred after the first 6 months, respectively, were 0.70 and 0.66 for major bleeding, 0.77 and 0.70 for major GI bleeding, and 0.74 and 0.71 for ICH. C-indices for the first year of follow-up and the entire follow-up period were 0.69 and 0.67 for major bleeding, 0.75 and 0.72 for major GI bleeding, and 0.72 and 0.72 for intracranial hemorrhage.

When we applied the various bleeding models to subgroups defined by indication for antithrombotic therapy, we found that c-index at 1 year of follow-up was lower for the major bleeding model applied to the coronary and peripheral artery disease population (c-index [95% CI], 0.66 [0.65–0.69]) as compared to the other two populations (0.73 [0.71–0.75] for atrial fibrillation and 0.73 [0.70–0.80] for ESUS). For the major GI bleeding model, the c-indices were similar across the populations. For the ICH model, the c-index was higher when applied to the ESUS population (0.82 [0.78–0.92]), especially when compared to the coronary and peripheral artery population (0.69 [0.66–0.79]; Table 4 ). When looking within these subpopulations, we found that c-indices at 1 year of follow-up were generally lower for the major bleeding model than for the site-specific bleeding models. This was most pronounced for the major GI bleeding model applied to the coronary or peripheral artery disease subpopulation (0.66 [0.65–0.69] for the major bleeding model vs. 0.75 [0.73–0.80] for the major GI bleeding model) and the ICH model applied to the ESUS population (0.73 [0.70–0.80] for the major bleeding model vs. 0.82 [0.78–0.92] for the ICH model; Table 4 ).

**Table 4:** Harrell's c-indices and 95% confidence intervals of models derived using the entire population for the prediction of major bleeding, major gastrointestinal bleeding, and intracranial hemorrhage, as applied, unmodified, to various subpopulations for prediction of various outcomes at 1 year

Model	Population			
	Overall	AF (AVERROES, RE-LY)	ESUS (NAVIGATE ESUS)	Coronary and peripheral artery disease (COMPASS)
Major bleed	0.69 (0.68, 0.71)	0.73 (0.71, 0.75)	0.73 (0.70, 0.80)	0.66 (0.65, 0.69)
Major GI bleed	0.75 (0.74, 0.78)	0.76 (0.73, 0.79)	0.73 (0.70, 0.85)	0.75 (0.73, 0.80)
ICH	0.72 (0.70, 0.79)	0.75 (0.70, 0.82)	0.82 (0.78, 0.92)	0.69 (0.66, 0.79)

Abbreviations: AF, atrial fibrillation; AVERROES, Apixaban Versus Acetylsalicylic Acid to Prevent Stroke in Atrial Fibrillation Patients Who Have Failed or Are Unsuitable for Vitamin K Antagonist Treatment; COMPASS, Cardiovascular Outcomes for People Using Anticoagulation Strategies; ESUS, embolic stroke of undetermined source; GI, gastrointestinal; ICH, intracranial hemorrhage; NAVIGATE ESUS, New Approach Rivaroxaban Inhibition of Factor Xa in a Global Trial versus ASA to Prevent Embolism in Embolic Stroke of Undetermined Source; RE-LY, Randomized Evaluation of Long-Term Anticoagulation Therapy.

### Calibration

**Fig. 2:**
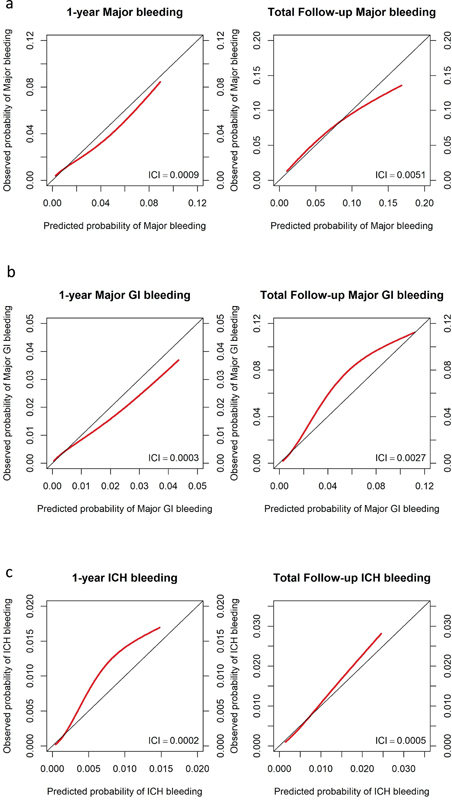
Calibration plots for major bleeding, major GI bleeding, and ICH models. ( **a** ) Major bleeding. ( **b** ) Major GI bleeding. ( **c** ) ICH. ICI—integrated calibration index.

Fig. 2 shows calibration plots and integrated calibration index for the major bleeding, major GI bleeding, and ICH models, with follow-up truncated at 1 year, and for the entire follow-up period. The model for major bleeding displayed some overprediction, whereas the models for major GI bleeding and ICH displayed some underprediction. Calibration was relatively similar whether truncating at 1 year or the entire follow-up period.

### Differences in Predictors Between Major GI Bleeding and ICH Models

When we produced new models to predict major GI bleeding and ICH which were forced to include all variables selected in the major bleeding, major GI bleeding, and ICH models described above, the hazard ratios of three variables had non-overlapping 95% CIs ( Table 2 ). For major GI bleeding and ICH respectively, the hazard ratios and 95% CIs for these variables were 0.93 (0.51–1.68) and 5.18 (2.67–10.03) for randomized treatment of warfarin vs. aspirin; 1.14 (0.98–1.32) and 2.21 (1.62–3.02) for the history of stroke at baseline; and 1.22 (1.04–1.43) and 0.79 (0.60–1.02) for baseline use of diuretic.

## Discussion

We examined the performance of bleeding risk prediction models according to the anatomical site of bleeding, time since initiation of antithrombotic therapy, and the underlying indication for antithrombotic therapy using a dataset of patients with coronary or peripheral artery disease, recent ESUS, or atrial fibrillation. There were three major findings: first, bleeding site-specific models provided better risk discrimination than the model which included major bleeding from any anatomical site; second, the performance of the models declined with duration of follow-up since starting antithrombotic therapy; and third, the performance of the models varied depending on the underlying indication for antithrombotic therapy.

Most previous bleeding prediction models were developed using datasets that included major bleeding from any anatomical site. 16 17 18 19 20 21 Few previous studies have separately examined risk prediction for different sites of bleeding. The QBleed algorithm separately predicted upper GI bleeding and ICH, but the performance of these models was not compared against the prediction of any major bleeding. 22

Our results demonstrating that models perform better when focused on individual sites of bleeding are not surprising because several risk factors for bleeding have been shown in prior studies to have different strengths of association with ICH and major GI bleeding. For example, the finding in our study that history of stroke and warfarin use were stronger predictors of ICH than of major GI bleeding is consistent with prior reports. 23 In contrast, the finding that lower diastolic blood pressure and diuretic use were predictors of GI bleeding but not of ICH was unexpected but in the context of contemporary trials with overall excellent blood pressure control this may reflect the effect of reduced perfusion in the splanchnic circulation. Others have reported that higher diastolic pressure was associated with ICH, 24 which suggests that diastolic blood pressure may help to differentiate between those at risk of intracranial versus GI bleeding. Overfitting is unlikely to explain the better discrimination of the site-specific models compared with the major bleeding model, since the site-specific models included fewer variables than the major-bleeding model, the optimism-corrected c-indices were similar to the uncorrected c-indices, and the calibration curves do not show patterns typical of overfitted models. The importance of separately examining the risk of bleeding by anatomic site is underscored by differences in prognosis; major upper GI bleeding is associated with in-hospital mortality of 10%, whereas ICH is associated with in-hospital mortality of over 30%. 25 26 GI bleeding and ICH account for about 75% of all major bleeding events in patients treated with antithrombotic therapy and their separate prediction could improve evaluation of the risks and benefits of antithrombotic therapies.

Discriminative performance of most previous bleeding prediction models is described at median follow-up of 1 or 2 years. 16 17 21 Our finding that bleeding risk discrimination declines over the first 2 years for major GI bleeding, and 1 year for ICH, limits the utility of most currently available risk prediction models beyond this time and suggests that separate tools are needed to predict risk during chronic therapy. This conclusion is supported by results of the COMPASS trial which found that excess major bleeding with the combination of rivaroxaban and aspirin compared with aspirin alone was largely confined to the first year after starting treatment. 27 To address this issue, time-varying covariates as used in the ATRIA bleeding model 19 and time-by-treatment interactions could be employed.

Most previously published models were derived using an atrial fibrillation population. 16 17 18 19 We unexpectedly found that major bleeding and ICH prediction model performance was worst when applied to patients with coronary and peripheral artery disease enrolled in COMPASS, which contributed 35% of bleeding events and nearly half of the patients in our dataset; bleeding models derived from other populations may not optimally predict bleeding for these patients.

### Strengths and Limitations

A strength of our approach is the use of a large dataset in which baseline characteristics and bleeding events were systematically collected using similar definitions. Our analyses also have limitations. Our models were derived from a population enrolled in randomized controlled trials, and our findings may not apply equally to a community population. Several potentially important variables were not available in all datasets. The duration of follow-up differed by indication for anticoagulation, and we were unable to explore the performance of our risk prediction model beyond 3 to 4 years. Our results are only applicable to the antithrombotic drugs and treatment indications included in our dataset. We were unable to account for neuroimaging markers of cerebral small vessel disease which have been established to be potent predictors of ICH in patients receiving anticoagulant treatment. Despite this, our ICH model applied to the ESUS population (c-index 0.82) performed well compared to the MICON-ICH model that included these neuroimaging markers in patients with prior history of ischemic stroke/TIA (optimism-corrected c-index 0.73). 28 We also included experimental treatments (rivaroxaban 5 mg bid in coronary and peripheral artery disease, rivaroxaban 15 mg once daily in ESUS) that are not approved for these indications. Our intent, however, was not to develop a new clinical risk prediction tool but rather to explore model performance in different contexts. We considered only first events in these analyses. We did this because many important predictors such as antithrombotic treatments are likely to change at the time of a bleeding or MACE event. Lastly, it is important to note that many of the variables that predict bleeding (i.e., older age, prior history of stroke, history of hypertension, etc.) are also predictors of heightened thrombotic risk and thus bleeding risk scores should not be used in isolation to withhold anticoagulation where otherwise indicated but are rather best used for prognostication and to identify and mitigate modifiable risk factors for bleeding complications.

## Conclusion

Our models performed better at identifying bleeding at specific anatomical sites than major bleeding at any site and at predicting bleeding earlier after initiation of treatment. Performance also varied according to subpopulation defined by indication for antithrombotic therapy. Future efforts to enhance bleeding risk prediction might consider the risk of major GI bleeding and ICH separately, and using datasets with a greater variety of antithrombotic agents and indications. Models that account for time since initiation of an antithrombotic medication, or strategies that allow for updating predictive variables, could result in improved prediction.

**List of Abbreviations**
AVERROES: Apixaban Versus Acetylsalicylic Acid to Prevent Stroke in Atrial Fibrillation Patients Who Have Failed or Are Unsuitable for Vitamin K Antagonist Treatment
bid: twice daily
CI: confidence interval
COMPASS: Cardiovascular Outcomes for People Using Anticoagulation Strategies
DOAC: direct oral anticoagulant
ESUS: embolic stroke of undetermined source
GI: gastrointestinal
ICH: intracranial hemorrhage
ISTH: International Society on Thrombosis and Haemostasis
MACE: major adverse cardiovascular events
NAVIGATE ESUS: New Approach Rivaroxaban Inhibition of Factor Xa in a Global Trial versus ASA to Prevent Embolism in Embolic Stroke of Undetermined Source
od: once daily
RE-LY: Randomized Evaluation of Long-Term Anticoagulation Therapy

## Notes

**Corrigendum:** A corrigendum has been published for this article (DOI: 10.1055/s-0044-1788303).
