# Corrigendum: Bleeding Risk Prediction in Patients Treated with Antithrombotic Drugs According to the Anatomic Site of Bleeding, Indication for Treatment, and Time Since Treatment Initiation

**DOI:** 10.1055/s-0044-1788303

**Published:** 2024-07-29

**Authors:** Vinai Bhagirath, Tanya Kovalova, Jia Wang, Lizhen Xu, Shrikant I. Bangdiwala, Martin O'Donnell, Ashkan Shoamanesh, Jackie Bosch, Rosa Coppolecchia, Tatsiana Vaitsiakhovich, Frank Kleinjung, Hardi Mundl, John Eikelboom

**Affiliations:** 1 Population Health Research Institute, Hamilton, Ontario, Canada; 2 McMaster University, Hamilton, Ontario, Canada; 3 University of Galway, Galway, Galway, Ireland; 4 Bayer US LLC, Whippany, New Jersey, United States; 5 Bayer AG, Berlin, Germany; 6 Bayer AG, Wuppertal, Germany

It has been brought to the publisher's attention that the author “Rosa Coppolecchia's” affiliation was changed to “Bayer US LLC, Whippany, New Jersey, United States” in the version of the above article in *TH Open* , published in Volume 8, Number 1 ( 10.1055/a-2259-1134 ). The updated authors list and the affiliations has been corrected as given above.

