# Supplementary material for: Bleeding Risk Prediction in Patients Treated with Antithrombotic Drugs According to the Anatomic Site of Bleeding, Indication for Treatment, and Time Since Treatment Initiation
Source: TH Open. 2024 Mar 18;8(1):e121–31. doi: 10.1055/a-2259-1134 (PMC10948265; doi:10.1055/a-2259-1134)
Supplement: Supplementary file 1 — Supplementary Material [file 10-1055-a-2259-1134-s23110047.pdf]

Supplementary Table S1 Summary of design of trials from which data were drawn

Trial	Study population	Study design	Intervention (s)	Control	*N*	Median follow-up
RE-LY	Atrial fibrillation	RCT	Dabigatran 150 mg bid; Dabigatran 110 mg bid	VKA	18, 113	2.0 y
AVERROES	Atrial fibrillation	RCT	Apixaban 5 mg bid (2.5 mg bid in certain patients)	ASA 81–324 mg daily	5,599	1.1 y
COMPASS	Coronary and peripheral artery disease	RCT	Rivaroxaban 2.5 mg bid and ASA 100 mg daily; Rivaroxaban 5 mg bid	ASA 100 mg daily	27, 395	1.9 y
NAVIGATE ESUS	Embolic stroke of undetermined source	RCT	Rivaroxaban 15 mg daily	ASA 100 mg daily	7,213	1.1 y

Abbreviations: ASA, acetyl salicylic acid; AVERROES, Apixaban Versus Acetylsalicylic Acid to Prevent Stroke in Atrial Fibrillation Patients Who Have Failed or Are Unsuitable for Vitamin K Antagonist Treatment; COMPASS, Cardiovascular Outcomes for People Using Anticoagulation Strategies; NAVIGATE ESUS, New Approach Rivaroxaban Inhibition of Factor Xa in a Global Trial versus ASA to Prevent Embolism in Embolic Stroke of Undetermined Source; RCT, randomized controlled trial; RE-LY, Randomized Evaluation of Long-Term Anticoagulation Therapy; VKA, vitamin K antagonist.

Supplementary Table S2 Baseline variables reported in the trials

Variable	COMPASS	NAVIGATE	RE-LY	AVERROES
Demographics				
Sex	x	x	x	x
Age	x	x	x	x
Ethnicity/race (Asian, black/African American, white/Caucasian, other)	x	x	x	x
Baseline physical measurements				
Systolic blood pressure	x	x	x	x
Diastolic blood pressure	x	x	x	x
Heart rate	x	x	x	x
Weight	x	x	x	x
Height	x	x	x	x
Hip circumference	x		x	x
Waist circumference	x		x	x
Baseline laboratory tests				
Creatinine	x	x	x	x
Blood urea nitrogen			x	
Hemoglobin	x	x	x	x
Platelets	x	x	x	x
Leukocytes	x	x	x	x
Aspartate aminotransferase	x	x	x	X
Alanine aminotransferase		x	x	x
Alkaline phosphatase		x	x	x
Total bilirubin		x	x	x
Direct bilirubin		x		
Sodium			x	
Potassium			x	
Chloride			x	
Phosphorus			x	
Protein			x	

 © 2024. The Author(s).

Supplementary Table S2 (*Continued*)

Variable	COMPASS	NAVIGATE	RE-LY	AVERROES
Albumin			x	
Lactate dehydrogenase			x	
Urate			x	
Total cholesterol			x	
Triglycerides			x	
Electrocardiogram			x	x
Baseline concomitant medications				
Aspirin	x	x	x	x
Clopidogrel	x	x	x	x
Prasugrel	x	x		
Ticagrelor	x	x		
Ticlopidine	x	x	x	x
Dipyridamole	x	x	x	x
Other antiplatelet	x	x	x	x
Parenteral anticoagulant	x	x	x	x
Rivaroxaban	x	x		
Apixaban	x	x		
Dabigatran	x	x		
Edoxaban		x		
Warfarin/vitamin K antagonist	x	x	x	X
Other anticoagulant	x	x		
Angiotensin receptor blocker	x	x	x	x
ACE inhibitor	x	x	x	x
Diuretic	x	x	x	x
Alpha blocker or other vasodilator	x	x	x	x
Calcium channel blocker	x	x	x	x
Beta blocker	x	x	x	x
Statin	x	x	x	x
Nonstatin lipid-lowering agent			x	x
COXII inhibitor			x	x
Other nonsteroidal anti-inflammatory drug	x	x	x	x
Insulin	x	x	x	x
Oral hypoglycemic	x	x	x	x
Proton pump inhibitor	x	x	x	x
H2 blocker			x	x
Digoxin			x	x
Amiodarone			x	x
Other antiarrhythmic			x	x
Selective serotonin reuptake inhibitor	x			x
Vitamins			x	
Herbal remedies			x	
Antibiotics			x	

(*Continued*)

 © 2024. The Author(s).

Supplementary Table S2 (*Continued*)

Variable	COMPASS	NAVIGATE	RE-LY	AVERROES
Questionnaires/assessments				
International Physical Activity Questionnaire	x			
Self-Administered Gerocognitive Exam	x	x		
Digit symbol substitution task	x			x
Montreal cognitive assessment	x	x		x
EuroQol 5D questionnaire	x	x	x	x
Trail-making test				x
1-minute semantic fluency (animal naming)				x
Medical history				
Stroke	x	x	x	x
Territory of qualifying stroke		x		
Treatment of qualifying stroke		x		
Severity of qualifying stroke (modified Rankin Scale, NIH stroke scale)		x		
Transient ischemic attack	x	x	x	x
Systemic embolism (non-CNS)			x	
Hypertension	x	x	x	x
Diabetes mellitus	x	x	x	x
Heart failure	x	x	x	x
Coronary artery disease	x	x	x	
Myocardial infarction	x	x	x	
Angina—stable or unstable	x			
Coronary atherectomy or percutaneous coronary intervention	x	x		
Coronary artery bypass grafting	x	x		
Carotid endarterectomy or stent		x		
Other cardiac arrhythmia			x	
Valvular heart disease			x	
Peripheral artery disease	x	x		x
Peripheral artery bypass surgery	x			
Percutaneous angioplasty	x			
Limb or foot amputation	x			
Intermittent claudication	x			
Asymptomatic carotid stenosis >50% or revascularization	x			
Aortic aneurysm	x			
Bioprosthetic heart valve present		x		
Permanent pacemaker or implantable cardiac defibrillator present		x	x	x
Venous thromboembolism		x		
Hyperlipidemia		x		
Tobacco use (current, former, and never)	x	x	x	x
Alcohol—more than five drinks per week	x		x	x
Cancer and site of primary	x	x	x	x
Bleeding requiring transfusion and site	x	x		
Prior vitamin K antagonist use			x	x
Bleeding while on oral anticoagulation			x	x

 © 2024. The Author(s).

Supplementary Table S2 (*Continued*)

Variable	COMPASS	NAVIGATE	RE-LY	AVERROES
Difficulty with INR control			x	x
Surgical history associated with gastrointestinal disease	x			
Peptic ulcer	x			
Diverticulitis	x			
Inflammatory bowel disease	x			
H. pylori	x			
Hemorrhoids	x			x
Daily or occasional nosebleeds				x
Liver disease	x	x		
Gilbert syndrome			x	x
History of fainting, falling, or fractures			x	x
History of seizures			x	
Ejection fraction	x	x	x	x
Qualifying atrial fibrillation episode			x	x
Type of atrial fibrillation (permanent, paroxysmal, and persistent)			x	x
Previous ablation			x	x
Previous cardioversion			x	x
Reasons why vitamin K antagonist is unsuitable				x
Social factors				
Health care costs (paid by self, public insurance, and private insurance)	x			
Driving status	x	x		
Employment status			x	x
Live alone				x
<12 y formal education	x	x		x

Abbreviations: ACE, angiotensin-converting enzyme; AVERROES, Apixaban Versus Acetylsalicylic Acid to Prevent Stroke in Atrial Fibrillation Patients Who Have Failed or Are Unsuitable for Vitamin K Antagonist Treatment; CNS, central nervous system; COMPASS, Cardiovascular Outcomes for People Using Anticoagulation Strategies; INR, international normalized ratio; NAVIGATE ESUS, New Approach Rivaroxaban Inhibition of Factor Xa in a Global Trial versus ASA to Prevent Embolism in Embolic Stroke of Undetermined Source; NIH, National Institutes of Health; RCT, randomized controlled trial; RE-LY, Randomized Evaluation of Long-Term Anticoagulation Therapy.
Note: x indicates variable collected in trial.

Supplementary Table S3 Number of patients with bleeding events per trial

First major bleeding event	COMPASS (N = 27,359)		RE-LY (N = 17,713)		NAVIGATE-ESUS (N = 7,195)		AVERROES (N = 5,546)		Total (N = 57,813)	
	N	%/y	*N*	%/y	*N*	%/y	*N*	%/y	*N*	%/y
Any major bleeding	688	1.3	1100	3.3	83	1.2	77	1.1	1,948	2.0
Intracranial bleeding	94	0.2	128	0.4	30	0.4	22	0.3	274	0.3
Major GI bleeding	272	0.5	393	1.1	30	0.4	22	0.3	717	0.7

Abbreviations: AVERROES, Apixaban Versus Acetylsalicylic Acid to Prevent Stroke in Atrial Fibrillation Patients Who Have Failed or Are Unsuitable for Vitamin K Antagonist Treatment; COMPASS, Cardiovascular Outcomes for People Using Anticoagulation Strategies; ESUS, embolic stroke of undetermined source; GI, gastrointestinal; NAVIGATE ESUS, New Approach Rivaroxaban Inhibition of Factor Xa in a Global Trial versus ASA to Prevent Embolism in Embolic Stroke of Undetermined Source; RE-LY, Randomized Evaluation of Long-Term Anticoagulation Therapy.

 © 2024. The Author(s).

Supplementary Table S4 Univariate analysis: Hazard ratios (95% confidence intervals) of variables for major bleeding, major GI bleeding, intracranial hemorrhage, and non-GI, nonintracranial bleeding

	Major bleeding		Major GIB		ICH	
	Hazard ratio	*p*-Value	Hazard ratio	*p*-Value	Hazard ratio	*p*-Value
Treatment arm (vs. aspirin alone)						
Warfarin	1.83 (1.30–2.57)	0.0005	0.90 (0.50–1.62)	0.72	5.11 (2.64–9.92)	<0.0001
DOAC, theraupetic dose	1.55 (1.13–2.13)	0.007	1.17 (0.68–2.02)	0.57	1.60 (0.92–2.81)	0.1
Rivaroxaban alone	1.56 (1.27–1.90)	<0.0001	1.66 (1.18–2.34)	0.003	1.50 (0.91–2.47)	0.11
Rivaroxaban + aspirin	1.78 (1.47–2.17)	<0.0001	2.47 (1.79–3.40)	<0.0001	1.11 (0.65–1.88)	0.7
Age (per 10 y increase)	1.72 (1.62–1.82)	<0.0001	1.98 (1.79–2.19)	<0.0001	1.66 (1.42–1.93)	<0.0001
Female	0.94 (0.85–1.03)	0.19	0.92 (0.78–1.09)	0.33	0.88 (0.67–1.14)	0.33
Race						
Caucasian	1.11 (1.00–1.22)	0.04	0.95 (0.81–1.11)	0.51	0.67 (0.53–0.86)	0.001
Asian	0.96 (0.85–1.09)	0.53	1.02 (0.83–1.24)	0.86	2.02 (1.56–2.64)	<0.0001
Black	1.43 (0.97–2.10)	0.07	1.63 (0.90–2.95)	0.11	1.51 (0.56–4.05)	0.41
Other	0.85 (0.74–0.97)	0.01	1.02 (0.84–1.25)	0.81	0.75 (0.52–1.08)	0.12
Weight (per 10 kg increase)	0.98 (0.95–1.00)	0.07	0.95 (0.92–1.00)	0.03	0.85 (0.79–0.91)	<0.0001
Height (per 10 cm increase)	0.99 (0.94–1.03)	0.55	0.96 (0.89–1.03)	0.27	0.94 (0.84–1.06)	0.34
BMI (per 10 units increase)	0.92 (0.85–1.00)	0.06	0.85 (0.74–0.99)	0.03	0.53 (0.41–0.68)	<0.0001
Baseline diastolic BP (per 10 units increase)	0.81 (0.78–0.85)	<0.0001	0.72 (0.67–0.78)	<0.0001	1.06 (0.94–1.18)	0.35
Baseline systolic BP (per 10 units increase)	1.00 (0.97–1.02)	0.87	0.97 (0.93–1.01)	0.2	1.04 (0.97–1.11)	0.3
Pulse pressure (per 10 units increase)	1.11 (1.07–1.14)	<0.0001	1.13 (1.07–1.18)	<0.0001	1.02 (0.94–1.11)	0.56
Heart rate (per 10 units increase)	0.98 (0.95–1.02)	0.3	0.98 (0.93–1.04)	0.51	0.97 (0.88–1.06)	0.46
Creatinine (per 10 µmol/L increase)	1.11 (1.09–1.14)	<0.0001	1.13 (1.09–1.17)	<0.0001	1.03 (0.96–1.12)	0.39
GFR (per 10 mL/min/1.73 m^3 increase)	0.86 (0.84–0.88)	<0.0001	0.82 (0.79–0.85)	<0.0001	0.86 (0.81–0.91)	<0.0001
Medical history						
Previous PAD	1.31 (1.14–1.51)	0.0001	1.30 (1.04–1.63)	0.02	0.76 (0.49–1.18)	0.21
Previous stroke	1.15 (0.99–1.33)	0.07	0.95 (0.73–1.24)	0.73	2.39 (1.76–3.26)	<0.0001
Previous TIA	1.16 (0.98–1.37)	0.09	1.18 (0.89–1.56)	0.26	1.06 (0.65–1.72)	0.81
HTN history	1.22 (1.09–1.37)	0.0006	1.25 (1.03–1.51)	0.02	1.18 (0.88–1.60)	0.27
Previous MI	1.20 (1.07–1.33)	0.001	1.33 (1.12–1.59)	0.001	1.06 (0.78–1.43)	0.72
Previous HF	1.02 (0.93–1.13)	0.65	1.21 (1.03–1.42)	0.02	0.86 (0.64–1.15)	0.32
Coronary artery disease	1.47 (1.31–1.65)	<0.0001	1.63 (1.35–1.97)	<0.0001	1.07 (0.77–1.48)	0.7
Cancer	1.53 (1.34–1.75)	<0.0001	1.35 (1.07–1.70)	0.01	1.35 (0.93–1.96)	0.12

 © 2024. The Author(s).

Supplementary Table S4 (*Continued*)

	Major bleeding		Major GIB		ICH	
	Hazard ratio	*p*-Value	Hazard ratio	*p*-Value	Hazard ratio	*p*-Value
Diabetes	1.18 (1.07–1.30)	0.001	1.16 (0.99–1.37)	0.06	0.96 (0.73–1.26)	0.76
Prerandomization medications						
Statin	1.09 (0.99–1.21)	0.09	1.16 (0.98–1.38)	0.08	0.78 (0.60–1.03)	0.08
Antiplatelet or aspirin	1.42 (1.27–1.59)	<0.0001	1.39 (1.16–1.68)	0.0004	1.32 (0.97–1.80)	0.07
Anticoagulant	1.11 (0.99–1.25)	0.08	1.09 (0.90–1.33)	0.37	1.19 (0.87–1.64)	0.27
PPI	1.31 (1.18–1.46)	<0.0001	1.31 (1.11–1.56)	0.002	1.26 (0.95–1.68)	0.11
NSAID	1.32 (1.11–1.56)	0.001	1.48 (1.14–1.93)	0.004	1.31 (0.83–2.07)	0.24
ACE or ARB	1.05 (0.96–1.16)	0.28	1.12 (0.95–1.31)	0.17	0.88 (0.69–1.13)	0.33
Beta blocker	0.93 (0.85–1.03)	0.16	0.91 (0.78–1.07)	0.25	0.85 (0.66–1.10)	0.22
Calcium channel blocker	1.11 (1.01–1.22)	0.03	1.08 (0.92–1.26)	0.34	1.04 (0.80–1.35)	0.76
Diuretic	0.86 (0.84–0.88)	<0.0001	0.82 (0.79–0.85)	<0.0001	0.87 (0.82–0.92)	<0.0001
> 12 y education	0.93 (0.81–1.07)	0.32	0.83 (0.66–1.04)	0.1	1.00 (0.72–1.39)	0.99
Smoking (vs. never smoking)						
Former	1.37 (1.25–1.51)	<0.0001	1.47 (1.25–1.72)	<0.0001	1.18 (0.91–1.52)	0.21
Current	1.09 (0.94–1.27)	0.26	1.13 (0.88–1.46)	0.33	1.01 (0.68–1.50)	0.95
>5 drinks of alcohol/wk	0.98 (0.89–1.08)	0.64	0.89 (0.76–1.05)	0.16	0.99 (0.76–1.29)	0.94

Abbreviations: ACE, angiotensin-converting enzyme; ARB, angiotensin II receptor blockers; BMI, body mass index; DOAC, direct oral anticoagulant; GFR, glomerular filtration rate; HF, Heart failure; HTN, hypertension; MI, myocardial infarction; NSAID, nonsteroidal anti-inflammatory drugs; PAD, peripheral artery disease; PPI, proton-pump inhibitors; TIA, transient ischemic attack.
Note: Univariate analysis by univariate Cox models. *p*-Values are from the Wald test used in the Cox models.

TRIPOD Checklist: Prediction Model Development

Section/Topic	Item	Checklist item	Page
Title and abstract			
Title	1	Identify the study as developing and/or validating a multivariable prediction model, the target population, and the outcome to be predicted.	1
Abstract	2	Provide a summary of objectives, study design, setting, participants, sample size, predictors, outcome, statistical analysis, results, and conclusions.	2
Introduction			
Background and objectives	3a	Explain the medical context (including whether diagnostic or prognostic) and rationale for developing or validating the multivariable prediction model, including references to existing models.	4
	3b	Specify the objectives, including whether the study describes the development or validation of the model or both.	4
Methods			
Source of data	4a	Describe the study design or source of data (e.g., randomized trial, cohort, or registry data), separately for the development and validation data sets, if applicable.	5
	4b	Specify the key study dates, including start of accrual; end of accrual; and, if applicable, end of follow-up.	5
Participants	5a	Specify key elements of the study setting (e.g., primary care, secondary care, general population) including number and location of centres.	S1
	5b	Describe eligibility criteria for participants.	S1
	5c	Give details of treatments received, if relevant.	S1
Outcome	6a	Clearly define the outcome that is predicted by the prediction model, including how and when assessed.	5–7
	6b	Report any actions to blind assessment of the outcome to be predicted.	5–6
Predictors	7a	Clearly define all predictors used in developing or validating the multivariable prediction model, including how and when they were measured.	10–11
	7b	Report any actions to blind assessment of predictors for the outcome and other predictors.	7-8
Sample size	8	Explain how the study size was arrived at.	5
Missing data	9	Describe how missing data were handled (e.g., complete-case analysis, single imputation, multiple imputation) with details of any imputation method.	8
Statistical analysis methods	10a	Describe how predictors were handled in the analyses.	7–8
	10b	Specify type of model, all model-building procedures (including any predictor selection), and method for internal validation.	7–8
	10d	Specify all measures used to assess model performance and, if relevant, to compare multiple models.	8–9
Risk groups	11	Provide details on how risk groups were created, if done.	n/a
Results			
Participants	13a	Describe the flow of participants through the study, including the number of participants with and without the outcome and, if applicable, a summary of the follow-up time. A diagram may be helpful.	S1,S9
	13b	Describe the characteristics of the participants (basic demographics, clinical features, available predictors), including the number of participants with missing data for predictors and outcome.	23–26
Model development	14a	Specify the number of participants and outcome events in each analysis.	23
	14b	If done, report the unadjusted association between each candidate predictor and outcome.	S10–16
Model specification	15a	Present the full prediction model to allow predictions for individuals (i.e., all regression coefficients, and model intercept or baseline survival at a given time point).	27–29

 © 2024. The Author(s).

TRIPOD Checklist: (*Continued*)

Section/Topic	Item	Checklist item	Page
	15b	Explain how to the use the prediction model.	n/a
Model performance	16	Report performance measures (with CIs) for the prediction model.	11–14
Discussion			
Limitations	18	Discuss any limitations of the study (such as nonrepresentative sample, few events per predictor, missing data).	17–18
Interpretation	19b	Give an overall interpretation of the results, considering objectives, limitations, and results from similar studies, and other relevant evidence.	15–18
Implications	20	Discuss the potential clinical use of the model and implications for future research.	18
Other information			
Supplementary information	21	Provide information about the availability of supplementary resources, such as study protocol, Web calculator, and data sets.	n/a
Funding	22	Give the source of funding and the role of the funders for the present study.	19

Abbreviation: CI, confidence interval.
Note: We recommend using the TRIPOD Checklist in conjunction with the TRIPOD Explanation and Elaboration document.

 © 2024. The Author(s).
